# Simultaneous Quantification of Dexamethasone and Cortisol for the Dexamethasone Suppression Test Using LC-MS/MS: Adaptation of a Commercial CE-IVD Steroid Assay

**DOI:** 10.1097/FTD.0000000000001389

**Published:** 2025-09-30

**Authors:** Regula Steiner, Marc Luginbühl

**Affiliations:** Institute for Clinical Chemistry, University Hospital and University of Zurich, Zurich, Switzerland.

**Keywords:** dexamethasone, cortisol, Cushing syndrome, steroids, LC-MS/MS

## Abstract

**Background:**

The dexamethasone suppression test (DST) is a key diagnostic tool for evaluating disorders of the hypothalamic–pituitary–adrenal (HPA) axis. Interpretation of DST results can be confounded by inadequate dexamethasone exposure due to pharmacokinetic variability or patient nonadherence. Simultaneous measurement of dexamethasone and cortisol by LC-MS/MS improves diagnostic accuracy by distinguishing true biological nonsuppression from insufficient drug exposure and prevents analytical interferences observed with cortisol immunoassays.

**Methods:**

A commercially available CE-IVD LC-MS/MS steroid panel (Chromsystems) was adapted for the simultaneous quantification of dexamethasone and cortisol in human plasma. The method was validated for linearity, accuracy, precision, selectivity, carry-over, matrix effects, and stability. Method comparison was performed using 26 clinical DST samples analyzed using immunoassay and external LC-MS testing. The proposed dexamethasone cutoff of 1.3 ng/mL (3.3 nmol/L) was evaluated in 62 patient samples.

**Results:**

The method demonstrated excellent linearity, with intra- and interassay accuracy and precision within ±15% for both analytes. No relevant carry-over or matrix effects were observed. Agreement with reference methods was 91% ± 6% for cortisol and 95% ± 7% for dexamethasone. Among the 62 DST samples, 48 showed appropriate suppression [cortisol <18.12 ng/mL (<50 nmol/L)] with dexamethasone levels above the cutoff. In contrast, 3 samples with insufficient suppression [cortisol >48.6 ng/mL (134.1 nmol/L)] had subthreshold dexamethasone levels, whereas 11 samples showed elevated cortisol levels despite sufficient dexamethasone exposure, indicating possible HPA axis dysfunction.

**Conclusions:**

The proposed rapid and robust LC-MS/MS method enables reliable, simultaneous quantification of dexamethasone and cortisol. The assay supports accurate DST interpretation by identifying cases of pharmacokinetic variance or nonadherence.

## BACKGROUND

Dexamethasone is a long-acting synthetic glucocorticoid with almost no mineralocorticoid activity.^1^ It has a biological half-life of approximately 36–72 hours and a relative glucocorticoid potency of 30 compared with cortisol.^2^ Due to its potent anti-inflammatory, immunosuppressive, and antiproliferative properties, dexamethasone is used pharmacologically for the treatment of inflammation, allergic and autoimmune disorders, leukemia, nausea, and vomiting, among other indications.^3–5^ Beyond its therapeutic applications, dexamethasone has widely been utilized as a diagnostic tool to assess disorders of the hypothalamic–pituitary–adrenal (HPA) axis. It is commonly employed to suppress endogenous cortisol production, thereby enabling evaluation of adrenal function or the pharmacodynamics of other glucocorticoids. For optimal suppression, dexamethasone is strategically administered when the HPA axis is most vulnerable to inhibition—typically at 11 pm. In this context, immediate-release 1-mg dexamethasone tablets are used as part of the 1-mg dexamethasone suppression test (DST) to ensure effective suppression of cortisol by the following morning.^4,6^ The test exploits the negative feedback mechanism by which exogenous dexamethasone suppresses endogenous cortisol secretion in individuals with an intact axis. An inadequate suppression response may indicate conditions such as Cushing syndrome, major depressive disorder, or pseudo-Cushing states.^3,7^

However, factors affecting dexamethasone availability or metabolism can significantly impact the interpretation of DST results. These include poor gastric absorption, rapid hepatic metabolism, altered protein binding, drug interactions, and patient nonadherence.^8,9^ In these scenarios, cortisol levels may remain elevated not due to HPA axis dysfunction, but rather due to insufficient dexamethasone exposure. Thus, measuring both dexamethasone and cortisol concentrations in DSTs enhances diagnostic accuracy by distinguishing true biological nonsuppression from pharmacokinetic failure.^10^

This study presents a novel approach for the simultaneous quantification of dexamethasone and cortisol using liquid chromatography-tandem mass spectrometry (LC-MS/MS) based on the adaptation of a commercially available Conformité Européenne – In Vitro Diagnostic (CE-IVD) steroid panel. Rapid, accurate, and simultaneous measurement of both analytes is essential to ensure reliable interpretation of DST results and identify cases in which test validity may be compromised by altered dexamethasone pharmacokinetics. The inclusion of cortisol measurement using Liquid Chromatography - Mass Spectrometry (LC-MS)/MS provides a plausibility check when prior determinations have been made using immunoassays and helps eliminate analytical interferences commonly associated with immunoassay-based methods such as electrochemiluminescence immunoassays (ECLIAs).^11^

## MATERIAL AND METHODS

### Method Development

The goal of this study was to develop and validate a method for the simultaneous quantification of dexamethasone and cortisol by adapting an existing CE-IVD LC-MS/MS steroid analysis method from Chromsystems (Gräfelfing, Germany). The focus was on creating a simple, rapid protocol that required no modifications to the existing LC-MS/MS system used for steroid profiling, such as changes in solvents or column exchanges. The 2 target analytes were integrated into a newly developed in-house LC-MS/MS method, with the chromatographic gradient optimized to achieve efficient and rapid separation. The novel method was thoroughly validated according to the M10. International Council for Harmonisation of Technical Requirements for Pharmaceuticals for Human Use (ICH) guideline on bioanalytical method validation and study sample analysis.^12^ Validation further included an internal method comparison for cortisol using ECLIA and an external comparison for both cortisol and dexamethasone using LC-MS/MS. Finally, the method was applied to analyze clinical DST samples. External quality assurance samples are available for cortisol; however, because we compared immunoassay and LC-MS methods, such samples were not included in method development. For dexamethasone, no external quality assurance samples are available, as it remains a niche analyte.

### Batch Design and QC Strategy

To provide a reliable measurement and monitor the robustness of the method, each analysis batch contained at least 1 set of 7 calibrator samples (K1–K7), a QC sample for each concentration (QC1–QC4), negative controls, and authentic samples that were used for method comparison.

### LC-MS/MS Analysis

**FIGURE 1.**
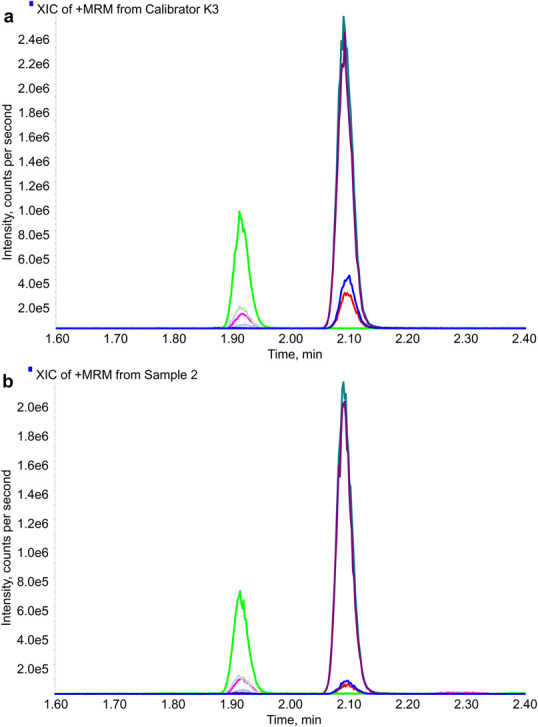
Representative extracted ion chromatograms for a calibrator sample spiked at K3 (A) and an authentic sample (B). The first peak represents cortisol, the second peak dexamethasone. Dexamethasone MRM1 (blue), MRM2 (red), dexamethasone-d_3_ MRM1 (purple), MRM2 (dark green), cortisol MRM1 (pink), MRM2 (gray), cortisol-d_3_ MRM1 (mint), MRM2 (bright green).

The LC-MS/MS system consisted of a PAL autosampler (CTC Analytics, Zwingen, Switzerland), an UltiMate 3000 system equipped with a degasser and 2 RS Pumps (Thermo Scientific, Waltham, MA), and a QTRAP 6500 system (Sciex, Framingham, MA). Liquid chromatography was performed using a MassChrom Steroids in Serum/Plasma analytical column (REF 72110) in conjunction with MassChrom Steroids in Serum/Plasma Mobile Phase A (REF 72011) and Mobile Phase B (REF 72002) from Chromsystems (Gräfelfing, Germany). Separation was initiated with 20% Mobile Phase B for 0.17 minutes, followed by a ramp to 99% Mobile Phase B, reached at 2 minutes. A 2-minute wash step was performed, after which the gradient was returned to 20% Mobile Phase B, and the column was reequilibrated for 0.5 minutes. The total runtime was 4.5 minutes, with a flow rate of 0.7 mL/min and an injection volume of 10 µL into a 5-µL loop (overfilling the loop). Electrospray ionization analysis was conducted in positive ionization mode, with the source temperature maintained at 400°C and the ion spray voltage set to 5500 V. The flow of curtain gas, gas 1, and gas 2 was set to 40 (arbitrary units). For each target analyte, 2 multiple reaction monitoring transitions were selected, and the declustering potential, entrance potential, collision energy, and cell exit potential were optimized by infusing standard solutions of each analyte. The target parameters are summarized in Table 1. A representative extracted ion chromatogram is shown in Figure 1.

**TABLE 1.** Optimized Mass Spectrometric Parameters for Analytes and Internal Standards

Analyte	ESI Mode	Q1 Mass [Da]	Q3 Mass [Da]	DP [V]	EP [V]	CE [V]	CXP [V]
Dexamethasone MRM1	+	393.3	373.1	30	15	13	12
Dexamethasone MRM2	+	393.3	355.1	30	15	13	17
Dexamethasone-d_3_ MRM1	+	396.4	358.1	30	15	13	17
Dexamethasone-d_3_ MRM2	+	396.4	376.4	30	15	13	12
Cortisol MRM1	+	363.3	121.2	80	10	25	35
Cortisol MRM2	+	363.3	96.8	80	10	25	30
Cortisol-d_3_ MRM1	+	366.5	97.0	80	10	25	30
Cortisol-d_3_ MRM2	+	366.5	121.1	80	10	25	35

### Sample Preparation

Calibration and QC samples were prepared by adding 10 µL of dexamethasone and cortisol standards (Toronto Research Chemicals, Toronto, Canada), diluted in Chromasolv methanol for high-performance liquid chromatography (≥99.9%, Honeywell, Seelze, Germany), to 240 µL of 0.9% NaCl (B. Braun Medical, Sempach, Switzerland). This resulted in final calibrator concentrations of 0.5, 1, 10, 50, 100, 250, and 500 ng/mL, and QC sample concentrations of 0.743, 7.42, 20.6, and 375 ng/mL. For authentic samples, 250 µL of heparin plasma was used. Extraction was performed in 1.5-mL Eppendorf Safe-Lock tubes by adding 10 µL of internal standard solution (1 mcg/mL), followed by 700 µL of methanol. Samples were vortexed at the maximum speed for 10 minutes using a Fisherbrand multi-tube vortexer (Fisher Scientific, Pittsburgh, PA), and then centrifuged at the maximum speed of 18′213*g* for 10 minutes on an Eppendorf 5427 R centrifuge. Supernatant (400 µL) was transferred into capped 75 × 12-mm tubes (Sarstedt, Nümbrecht, Germany) and evaporated under nitrogen (2.2 mL/min) in a TurboVap (Biotage, Uppsala, Sweden) at a temperature of 26°C. The dried residue was reconstituted in 100 µL of methanol and transferred into 0.3-mL polypropylene autosampler vials (Chromoptic, Courtaboeuf Cedex, France). If a larger LC-MS/MS sample loop is available, direct injection of 20 µL of supernatant without prior evaporation and concentration is also feasible.

## RESULTS

### Linearity

Linearity was established during 6 validation series, in which the calibrators K1–K7 were measured. A linear calibration model with 1/× weighting was used. The correlation coefficient (r) for the quantifier signal was 0.9996 ± 0.0005 (range: 0.9985–1.000) for dexamethasone and 0.9998 ± 0.0001 (range: 0.9996–0.9999) for cortisol.

### Accuracy and Precision

Accuracy and precision were investigated during the validation process by analyzing spiked samples at QC concentrations, both in intraday 1 × 6 and interday 6 × 1 designs (Table 2). Accuracy and precision for QC samples remained within the required ±15% during intra-assay and interassay evaluation. The lower limit of quantification was defined as the concentration of calibrator K1 and assessed for accuracy and precision using 6 replicate injections, with results consistently remaining within the ±15% acceptance criteria. A lower concentration was not considered, as it would not be clinically relevant or practical for the intended application of the method.

**TABLE 2.** Intra-assay and Interassay Accuracy and Imprecision, Assessed Using Intraday (1 × 6) and Interday (6 × 1) QC Analysis, Based on Quantifier Evaluation

Analyte	Concentration [ng/mL]	Intra-Assay Accuracy [%, n = 6]	Interassay Accuracy [%, n = 6]	Intra-Assay Imprecision [%, n = 6]	Interassay Imprecision [%, n = 6]
Dexamethasone MRM1	0.5	107.7	97.0	7.10	4.2
	0.743	91.5	89.4	6.3	6.5
	7.42	91.0	98.7	2.0	5.5
	20.6	85.1	92.8	2.2	12.1
	375	93.5	100.3	2.0	4.7
Cortisol MRM1	0.5	109.5	98.2	9.6	9.5
	0.743	94.8	93.9	5.1	12.2
	7.42	95.4	100.1	1.9	3.5
	20.6	96.0	95.8	1.5	12.5
	375	96.4	101.2	2.0	2.8

### Selectivity

The chromatographic conditions were optimized to achieve baseline separation of both analytes, ensuring accurate and reliable quantification (Fig. 1). During method evaluation, no interfering peaks were observed within the retention time window of the target analytes, indicating high selectivity and specificity of the analytical method.

### Carry-Over

Carry-over was evaluated in each analytical run by injecting a methanol blank immediately after injection of the highest calibrator concentration (K7, 500 ng/mL) and found to be negligible. The same result was observed after 6 consecutive injections of the most highly concentrated QC sample (QC4).

### Matrix Effect

Matrix effects were assessed by pooling 23 routine samples exhibiting icteric, hemolytic, and/or lipemic characteristics into 11 combined samples. These pooled samples were analyzed both with (QC2, 7.42 ng/mL) and without addition of analytes, and the signal increase between spiked and unspiked samples was then evaluated. No matrix effects were observed when using dexamethasone-d_3_ MRM1 as the internal standard. However, using dexamethasone-d_3_ MRM2 resulted in apparent matrix effects of 25%–30%.

### Sample Stability

Sample stability was assessed in 6 authentic samples stored at room temperature for periods of 3 days and 2 weeks. Cortisol remained stable at both time points, with 102.74 ± 3.47% (range: 96.43%–106.34%) of the initial concentration after 3 days and 102.12 ± 5.39% (range: 96.4%–112%) after 14 days. In contrast, dexamethasone exhibited reduced stability, with 87.45 ± 4.61% (range: 79.91%–92.52%) of the initial concentration after 3 days, and 53.45 ± 16.55% (range: 37.3%–87.1%) after 14 days. When stored in the fridge at approximately 4°C, both analytes remained stable for over 2 weeks, with cortisol at 106% ± 2.19% (range: 102%–109%) and dexamethasone at 108.67 ± 2.21% of the initial concentration (range: 104%–111%). After long-term storage in the freezer (approximately −20°C) for 10 months, 4 samples were reanalyzed. Dexamethasone showed a recovery of 80.58 ± 0.03% (range: 75.72%–83.68%), whereas cortisol showed a recovery of 89.81 ± 0.10% (range: 79.17%–105.30%). Furthermore, samples remained stable throughout 2 freeze–thaw cycles and during international shipment for method comparison.

### Method Comparison

For method comparison, 26 authentic samples from DSTs were analyzed. These samples had previously undergone routine cortisol measurement via ECLIA on a Cobas 8000 system (Roche Diagnostics, Rotkreuz, Switzerland) using the Elecsys Cortisol II Assay from Roche. The results were used for in-house comparison. The mean agreement between ECLIA and LC-MS/MS for cortisol was 109% ± 6% (range: 99%–125%).

**FIGURE 2.**
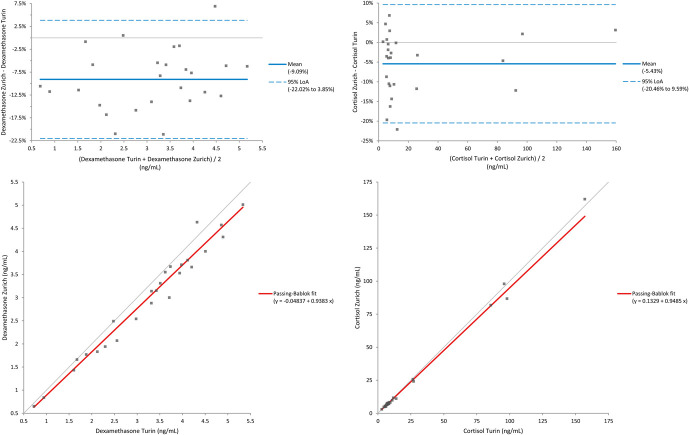
Bland–Altman and Passing–Bablok regression analyses comparing LC-MS/MS results between laboratories for dexamethasone (left, r = 0.984) and cortisol (right, r = 0.998).

The same samples were subsequently sent to the Laboratory of Endocrinology, Diabetology, and Metabolism at Molinette Hospital in Turin, which kindly agreed to perform a comparative analysis of both cortisol and dexamethasone using LC-MS. The results of this method comparison are summarized in Table 3. The mean agreement between the 2 laboratories was 91% ± 6% for cortisol (range: 81%–107%) and 95% ± 7% for dexamethasone (range: 80%–107%). Details on Passing–Bablok regression and Bland–Altman analysis are shown in Figure 2.

**TABLE 3.** Method Comparison; Cortisol and Dexamethasone Measurements in Zurich and Turin

Sample No.	Zurich/Switzerland			Turin/Italy			
	Cortisol ECLIA	Cortisol LC-MS	Dexamethasone LC-MS	Cortisol LC-MS	Dexamethasone LC-MS	Deviation Cortisol LC-MS	Deviation Dexamethasone LC-MS
	ng/mL	ng/mL	ng/mL	ng/mL	ng/mL	%	%
1	5.07	5.05	3.55	5.51	3.62	98	92
2	24.60	25.70	4.63	26.55	4.32	107	97
3	5.79	6.40	1.66	6.43	1.67	99	100
4	5.07	5.02	3.15	6.12	3.42	92	82
5	7.24	8.28	2.49	8.51	2.48	101	97
6	10.90	11.10	4.31	13.86	4.90	88	80
7	5.07	5.53	2.54	5.49	2.98	85	101
8	78.20	81.80	0.65	85.70	0.72	90	95
9	2.53	2.96	2.88	2.95	3.31	87	100
10	152.00	162.00	3.53	157.03	3.94	90	103
11	6.88	7.46	3.00	6.97	3.71	81	107
12	86.90	86.70	1.43	97.98	1.60	89	88
13	9.05	9.77	2.07	10.87	2.56	81	90
14	6.88	7.14	1.77	8.41	1.88	94	85
15	11.20	11.70	4.00	11.72	4.51	89	100
16	3.98	4.72	3.14	4.50	3.32	95	105
17	93.76	97.90	0.84	95.82	0.94	89	102
18	5.07	6.32	5.01	6.44	5.33	94	98
19	5.79	6.57	4.57	7.30	4.86	94	90
20	5.43	6.35	1.83	6.61	2.12	86	96
21	7.60	8.15	3.81	9.41	4.11	93	87
22	6.88	7.58	3.71	7.36	3.98	93	103
23	23.17	24.10	3.67	27.12	3.73	98	89
24	6.88	7.83	3.66	8.14	4.20	87	96
25	6.52	7.17	3.31	8.01	3.51	94	90
26	4.71	5.30	1.94	5.49	2.30	84	97

### Evaluation of Cutoff Concentration

The proposed dexamethasone cutoff concentration of 1.3 ng/mL (3.3 nmol/L) for the DST, measured using LC-MS/MS, was evaluated in samples from 62 patients.^10,13^ Among them, 48 patients had cortisol concentrations below the target value of 18.12 ng/mL (<50 nmol/L), in combination with dexamethasone concentrations above the defined cutoff. In this subgroup, the mean dexamethasone concentration was 3.92 ± 1.76 ng/mL (range: 1.72–10.90 ng/mL), whereas cortisol concentrations averaged 8.16 ± 2.95 ng/mL (range: 2.96–14.70 ng/mL). Three patients exhibited insufficient cortisol suppression, with elevated cortisol levels of 76.10 ± 20.53 ng/mL (range: 48.60–97.90 ng/mL), accompanied by dexamethasone concentrations below the cutoff (mean: 0.747 ± 0.11 ng/mL; range: 0.63–0.90 ng/mL). In the remaining 11 patients, dexamethasone concentrations were above the cutoff (mean: 3.84 ± 1.69 ng/mL; range: 1.42–7.29 ng/mL), yet cortisol levels remained elevated, averaging 88.06 ± 65.38 ng/mL (range: 19.20–537.98 ng/mL). Six samples showed clearly elevated cortisol concentrations >72.5 ng/mL (>200 nmol/L), whereas the other 5 were near the target threshold, with values ranging from 19.2 to 28.4 ng/mL (53.0–78.4 nmol/L). Cortisol concentrations below the target value of 18.12 ng/mL (<50 nmol/L) in combination with dexamethasone concentrations below the cutoff concentration of 1.3 ng/mL (3.3 nmol/L) were not observed.

## DISCUSSION

This study demonstrates the successful adaptation of a commercially available CE-IVD steroid panel for the simultaneous quantification of dexamethasone and cortisol using LC-MS/MS. The proposed method enables a reliable and clinically relevant assessment of DST outcomes. By modifying an existing analytical setup for steroid analysis, we developed a robust method with excellent analytical performance that is suitable for routine diagnostics without requiring major changes.

Method validation confirmed key performance criteria, including linearity, accuracy, precision, selectivity, carry-over, matrix effects, and analyte stability. Linearity was maintained across the full range of clinically relevant concentrations for both analytes, and intra-/interassay variability remained within ±15%, in line with international bioanalytical guidelines. Particularly noteworthy was the absence of significant matrix effects when using the primary multiple reaction monitoring transition of dexamethasone-d_3_ as an internal standard, which supports the robustness of the method across a wide spectrum of sample conditions, including hemolytic, icteric, and lipemic matrices.

A critical finding of the method comparison was the high level of agreement between our LC-MS/MS results and those from external reference laboratories, with average concordance of 91% ± 6% for cortisol and 95% ± 7% for dexamethasone. This highlights the assay's reliability and confirms its suitability for use in clinical decision making. The clinical evaluation of the dexamethasone cutoff of 1.3 ng/mL (3.3 nmol/L) reinforced its utility in distinguishing between true nonsuppression and insufficient drug exposure. Among the 62 DST samples, 48 (77%) showed appropriate cortisol suppression in the presence of adequate dexamethasone levels, consistent with a normal HPA axis response. In contrast, 3 samples with high cortisol and subthreshold dexamethasone concentrations likely reflected incomplete drug absorption, fast metabolism, or nonadherence rather than endogenous cortisol overproduction. Without concurrent dexamethasone quantification, these cases might have been misinterpreted as pathological nonsuppression.

The subgroup of 11 patients with elevated cortisol levels, despite adequate dexamethasone levels, is of particular diagnostic interest. Although 5 samples exhibited borderline cortisol elevations, 6 showed clearly pathological cortisol concentrations [>72.5 ng/mL (200 nmol/L)], which are strongly suggestive of Cushing syndrome or other forms of HPA axis dysregulation. In such cases, simultaneous measurement allows clinicians to confidently exclude low dexamethasone exposure and proceed with further endocrine work-up.

Interestingly, no individuals fell into a theoretical fourth category characterized by both low cortisol and dexamethasone levels. The absence of this phenotype in our cohort supports the validity of the dexamethasone cutoff, as it suggests that cortisol suppression does not occur in the presence of subthreshold dexamethasone concentrations.

Importantly, our stability experiments underscore the critical importance of stringent preanalytical handling, particularly for dexamethasone. Although cortisol remained stable at room temperature for up to 14 days, dexamethasone showed substantial degradation, with levels declining by nearly 50% over the same period. Although both analytes remained stable for at least 2 weeks when refrigerated at 4°C, prolonged storage at −20°C revealed a gradual decline, especially for dexamethasone, which lost nearly 20% of its initial concentration over 10 months. These findings emphasize the necessity of cool-chain logistics and prompt processing, especially in multicenter studies or routine clinical settings where delays in transport or analysis may compromise analyte integrity—most notably for dexamethasone.

A potential limitation of the study is the relatively small sample size in the clinical evaluation cohort, especially in the subgroup with insufficient suppression. However, the consistent patterns observed across patient categories provide a solid foundation for clinical application, and further validation in larger cohorts is ongoing. Another limitation is the use of plasma instead of serum, which, although practical and validated here, may require harmonization when comparing the results to those from laboratories that use serum-based reference ranges.

In summary, this study provides a clinically validated LC-MS/MS method that allows for the simultaneous and accurate quantification of dexamethasone and cortisol in plasma. The method enhances DST interpretation by accounting for pharmacokinetic variability and patient adherence, reducing the risk of diagnostic errors. Incorporating dual-analyte quantification into routine DST workflows represents a significant improvement in the diagnostic evaluation of HPA axis disorders, particularly with respect to Cushing syndrome.

## CONCLUSIONS

We present a validated LC-MS/MS method for the simultaneous quantification of dexamethasone and cortisol, adapted from a commercial CE-IVD steroid panel, that is suitable for routine clinical use in the context of the DST. The assay enables reliable identification of insufficient dexamethasone exposure, thereby improving DST interpretation and potentially reducing diagnostic errors in endocrine evaluations. Its rapid run time and analytical robustness make it a valuable tool for modern clinical laboratories. Future work should aim at establishing diagnostic thresholds across diverse clinical contexts and exploring the method's integration into broader endocrinological test panels.
